# Aggregation-induced emission spectra of triphenylamine salicylaldehyde derivatives *via* excited-state intramolecular proton transfer revealed by molecular spectral and dynamics simulations†

**DOI:** 10.1039/d1ra07388e

**Published:** 2021-11-18

**Authors:** Qing Zhang, Yuanyuan Li, Zexing Cao, Chaoyuan Zhu

**Affiliations:** State Key Laboratory of Physical Chemistry of Solid Surfaces, Fujian Provincial Key Laboratory of Theoretical and Computational Chemistry, College of Chemistry and Chemical Engineering, Xiamen University Xiamen 360015 China zxcao@xmu.edu.cn; Department of Applied Chemistry, Institute of Molecular Science, National Chiao-Tung University Hsinchu 30010 Taiwan cyzhu@mail.nctu.edu.tw; Department of Applied Chemistry, Center for Emergent Functional Matter Science, National Yang Ming Chiao Tung University Hsinchu 30010 Taiwan

## Abstract

Aggregation-induced emission (AIE) spectra accompanied by excited state intramolecular proton transfer (ESIPT) for two triphenylamine salicylaldehyde derivatives (namely, TS and TS-OMe) are investigated by performing molecular spectral and dynamics simulations associated with the hybrid quantum mechanics/molecular mechanics (QM/MM) at the quantum level of the time-dependent density functional theory. The simulated emission spectral peaks and Stokes' shifts are in good agreement with the experimental results for both TS and TS-OMe. Furthermore, the AIE spectral mechanisms are well explained to be associated with the ESIPT processes for both TS and TS-OMe monomers in the aggregated crystal state, while the AIE spectra mechanism for the TS-OMe (TS) dimer is accompanied by intermolecular charge-transfer excitation process. Besides, the TS dimers also contributed to the AIE mechanisms in the crystal with the intermolecular charge-transfer from one monomer to another. In addition, the TS dimers are contributed to the AIE mechanisms in the crystal with the intermolecular charge-transfer from one monomer to another. On the other hand, simulated emission spectra for both the TS and TS-OMe monomers in acetonitrile solution are involved in mixed emission with and without the ESIPT process, as interpreted by nonadiabatic molecular dynamics simulation. It is also briefly addressed that the emission spectra in the solution are weak and enhanced in the crystal. The present study provides a great physical insight into the design of highly efficient AIE compounds.

## Abstract 2

The emission spectra of triphenylamine salicylaldehyde derivatives are weak in solution and strong in the crystal, as interpreted by molecular spectral and dynamics simulations.

## Graphical abstract

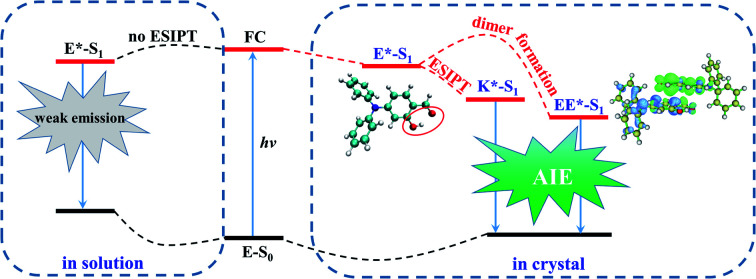

## 1. Introduction

Organic luminescent materials have attracted great attention for several decades because of their varied applications in organic light-emitting diodes (OLEDs), bioimaging, and chemical sensing.^1–3^ Generally speaking, organic luminescent materials exhibit a remarkably high luminescence efficiency in dilute solvents but their efficiency is significantly reduced in the solid state, mainly due to the formation of aggregation-caused quenching (ACQ). However, most organic luminescent materials must be fabricated in the solid state in order to enable their practical applications; thus, ACQ is a frequently experienced obstacle in the development of efficient organic luminescent materials.^4–6^ Fortunately, in 2001, Luo *et al.*^7^ proposed aggregation-induced emission (AIE), an intriguing photophysical phenomenon in which the organic luminescent materials exhibit faintly or negligible luminescence efficiency in dilute solvents; however, its efficiency is significantly enhanced in the aggregated or solid state. The AIE discovery has led to the resolution of the conventional problem encountered in ACQ, and thus, AIE has been greatly improved for the development of advanced organic luminescent materials.^8–11^ Since then, great efforts have been devoted for developing AIE materials and various AIE-accompanied mechanisms have been proposed to enhance the luminescence efficiency, such as J-aggregate formation,^12^ restricted access to the conical intersection (RACI),^13,14^ excimer emission,^15–17^ and excited-state intramolecular proton transfer (ESIPT).^18–21^

In the recent years, organic luminescent materials based on the coupling of AIE and ESIPT mechanisms have drawn widespread attention in bioimaging due to their high efficiency emission in the solid state and large Stokes' shifts.^22,23^ Generally speaking, ESIPT is a four-level photochemical process that takes place in the strong intramolecular hydrogen bonds transfer from the hydrogen bond donor (–OH and –NH_2_) to the hydrogen bond acceptor (=N– and C=O) through the singlet or triplet excited states (*i.e.*, enol (E) → excited enol (E*) → excited keto (K*) → keto (K));^24–29^ thus, it results in an interesting photoinduced enol–keto tautomerization. As a result, the emission spectrum band emitted from the K* isomer has almost no overlap with the absorption spectrum band produced from the E isomer, which can prevent the unwanted self-absorption in photochemistry and increase the emission efficiency. Liu and coworkers^22^ developed organic luminescent materials from an investigation of AIE combined with the ESIPT mechanism; since then, numerous studies have been reported on the “AIE + ESIPT” coupling schemes that have already become a hot topic in bioimaging applications.^30,31^

In particular, in 2020, Zhao and coworkers^32^ designed and synthesized three triphenylamine salicylaldehyde (TS) derivatives, namely, TS, TS-OMe (methoxy-substituted TS), and TS-NMe_2_ (dimethylamine-substituted TS), and all three showed AIE and ESIPT characteristics in the aggregated solid state. Their experiment showed that the TS derivatives in the aggregated solid state exhibited intense fluorescence emission and Stokes' shifts larger than 140 nm wavelength. Therefore, they speculated that this must be due to the ESIPT mechanism. The three TS derivatives in the aggregated solid state can be beneficial for *in vivo* imaging. In contrast, the three TS derivatives in dilute solutions exhibited faint emission; thus, they speculated that this weak emission originated from the E* isomers without involving the ESIPT mechanism.

Two of the three TS derivatives with higher fluorescence efficiencies, namely, TS and TS-OMe, are chosen in the present work for interpreting their spectacular photochemical spectra and dynamics in both acetonitrile solution and the aggregated solid state, and we explain using the AIE plus ESIPT mechanisms why the emission is suppressed in the solution but enhanced in the aggregated solid state from computational quantum chemistry calculations. Based on the time-dependent density functional theory (TD-DFT) method in a tutorial review article,^33–36^ the electronic structures and potential energies in the excited states for TS and TS-OMe in acetonitrile solution and the aggregated states are firstly simulated. The hybrid quantum mechanics/molecular mechanics (QM/MM) calculation with two layers ONIOM model^37^ are employed for simulation in the aggregated states. Secondly, we construct the potential energy surfaces of the ground (S_0_) and the lowest excited (S_1_) states in both the solution and crystal phases to explore their ESIPT mechanisms, followed by the nonadiabatic molecular dynamics simulation with on-the-fly trajectory surface hopping approaches^38–44^ for the TS and TS-OMe monomers. Furthermore, we quantitatively analyze the intermolecular interactions for the selected TS and TS-OMe dimers extracted from their experimental crystal phases using the energy decomposition analysis (EDA)^45^ plus the classical molecular force field (FF)^46,47^ in the solid state. Finally, we investigate the AIE spectra for the TS and TS-OMe dimers in comparison with their AIE spectra in order to deeply understand their emission nature in the solid state.

## 2. Computational details

**Fig. 1:**
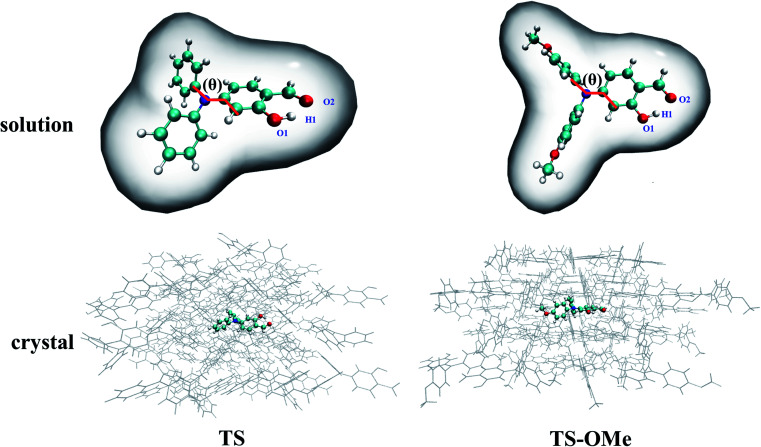
Computational sketches with the PCM model in acetonitrile solution and the QM/MM model in the aggregated crystal for TS and TS-OMe, respectively.

The BMK (Boese–Martin for Kinetics) hybrid functional^48^ in the TD-DFT method with the 6-31G(d,p) basis sets^49^ was tested for its good reproduction of experimental peak positions for both the absorption and emission spectra in both the solution and crystal phases. We utilize the (TD)-BKW/6-31G(d,p) method for optimizing the electronic structures and calculating the excited-state potential energies throughout the present work, unless otherwise specified. (TD)-BMK functional plus linear-response (LR)^50^ formally polarizable continuum scheme (PCM)^51^ is employed for calculating the geometries and potential energies of the TS and TS-OMe molecules in acetonitrile solution, as shown in the upper panel of Fig. 1. The initial geometry structures of the ONIOM calculations were constructed by extracting a 57-molecule cluster (2109 atoms in total) and a 48-molecule cluster (2160 atoms in total) from the experimental X-ray crystal structures for TS and TS-OMe, respectively. The TS (TS-OMe) molecule in the middle was set as the QM region and simulated by high-level QM calculations, while the surrounding 56 (47) molecules were treated as the environmental MM molecules and simulated by low-level universal force field (UFF). (TD)-BMK functional plus a two-layered ONIOM model with QM/MM scheme is employed for calculating the geometries and potential energies of the TS and TS-OMe molecules in the aggregated solid state, as shown in the lower panel of Fig. 1, in which TS and TS-OMe molecules embedded in the center are computed by the QM method and the surrounding MM molecules are modelled by the universal force field (UFF) method^52^ with the charge equilibration (QEQ) approach.^53^ A detailed simulation description of the computational procedure is given as follows. Especially in the optimization, only the central QM molecules can vary its geometry structures in both the ground and excited states, while the surrounding MM molecular geometries are frozen as the solid environment. Due to the large number of atoms in the MM regions (2072 atoms for TS and 2115 atoms for TS-OMe), only the Cartesian coordinates of the atoms within the QM regions in both the S_0_ and S_1_ states are given in Tables S1–S4 (ESI†). In addition, the surrounding MM molecules are simulated as the electrostatic potential embedded in the QM simulation, which plays a role in describing the electrostatic interactions between the central QM molecules and the surrounding MM molecules (ONIOM-EE).^54^ This ONIOM-EE strategy efficiently balances the computational cost and accuracy in calculation. The initial geometry structures of the QM/MM molecules are constructed from the experimental X-ray crystal structures by extracting all molecules within the 10 Å radius around the QM molecules. The all-equilibrium geometries of the TS and TS-OMe molecules in both the ground and excited states are verified as the local minima on the potential energy surfaces by carrying out normal-mode frequency calculation in the absence of imaginary frequency. Considering notable hydrogen bonds coming from the intramolecular interaction in the TS and TS-OMe monomers, we expect that there exist significant intermolecular interactions in the TS and TS-OMe dimers; thus, the dispersion-corrected functional with the Grimme's DFT-D3 correction^55,56^ is utilized. All QM/MM and PCM calculations are performed using the Gaussian 16 program.^57^

In order to deeply understand the AIE + ESIPT mechanisms speculated in the experimental emission spectra of the TS and TS-OMe molecules, we also perform trajectory surface hopping molecular dynamics simulation for the TS and TS-OMe monomers up to the excitation to the S_1_ excited states on the on-the-fly TD-BMK/6-31G(d) potential energy surfaces. We employ the global nonadiabatic switching algorithm^43^ for switching the on-the-fly trajectory from the excited state to the ground state potential energy surfaces. The initial coordinates and velocities of all the sampling trajectories are generated by stochastic sampling from the Wigner distributions transferred from the vibrational normal-mode coordinates and the momentums on the S_0_ state potential energy surface. The velocity Verlet algorithm^58^ is employed for the numerical integration of Newton's equations of motion for all the atoms with a time stepsize of 0.5 fs and a maximum simulation time of 1.5 ps. For each molecule, a total of 27 sampling trajectories are simulated. We unitize the interface Gaussian 16 program coded in the newly modified Newton-X^44,59^ package for performing trajectory surface hopping molecular dynamics simulation.

## 3. Results and discussion

Double-well potential energy surface in relation to the two local minima at K* and E* on excited-state S_1_ are found by optimization with the (TD)BMK/6-31G(d,p) + PCM method in acetonitrile solution as well as with the ONIOM-EE((TD)BMK/6-31G(d,p)):UFF method in the aggregated crystal state for both the TS and TS-OMe molecules. However, only single-well potential energy surface in relation to the global minimum at E form on the ground-state S_0_ are found by optimization in both acetonitrile solution and in the aggregated crystal state. This is because the proton transfers in the S_0_ state from hydrogen-bonded K to E form with no potential energy barrier; thus, only the E form hydrogen-bond on the ground state exists. The key geometry parameters related to hydrogen bonds H1–O1 and H1–O2, and dihedral angle *θ* (seeing Fig. 1 for definition), and the relative energies are summarized in Table S5 (ESI†). The present calculations for the TS and TS-OMe molecules agree well with experimentally measured E-form electronic structures in the crystal.^32^ The dihedral angle *θ*, which is defined as in between the triphenylamine and salicylaldehyde molecular planes, shows more distortion in solution than in the crystal for both TS and TS-OMe in the S_0_ and S_1_ states. Especially in the excited-state S_1_, the *θ* changes are restricted in the crystal due to the steric hindrance and intermolecular interactions induced by the surrounding environmental molecules. In the ground state S_0_ (only existing E-form), the bond length of H1–O2 in solution is 1.78 Å for both the TS and TS-OMe molecules, which indicates a typical intramolecular hydrogen bond, while in the crystal, it is 1.77 Å and 1.75 Å for TS and TS-OMe, respectively, which shows a typical enhanced intramolecular hydrogen bond. However, on the excited state S_1_ (both E* and K* forms exist), the H1–O2 bond in the E* form in both the solution and the crystal is shorter than that in its corresponding E form for TS and TS-OMe, which indicates a typical excited-state enhanced intramolecular hydrogen bond. As shown in Table S5 (ESI†), the relative energies of the E* forms are lower than their corresponding K* form energies in solution both for TS and TS-OMe. Besides, in the crystal, the E* form also has a lower energy for TS, while the K* form has a more stable structure with a lower energy of 1.7 kcal mol^−1^ for the TS-OMe isomer, indicating the possibility of the ESIPT process.

**Fig. 2:**
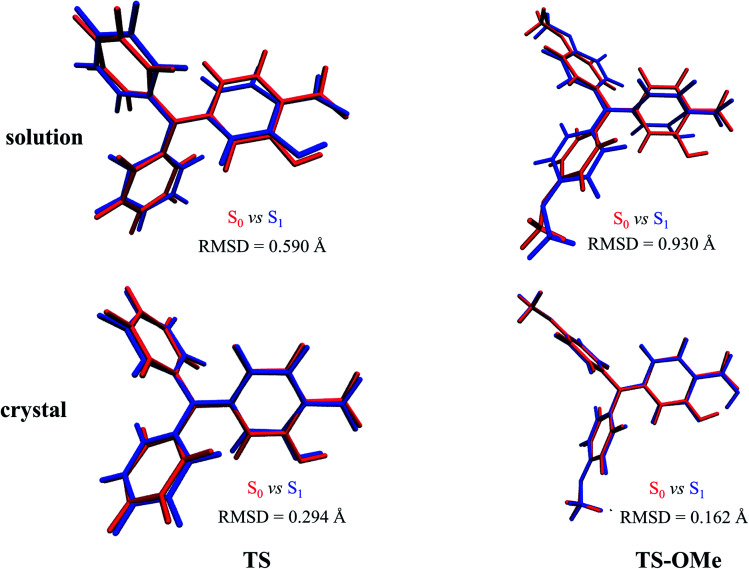
Geometry differences between S_0_ (red) and S_1_ (blue) for TS and TS-OMe monomers in both acetonitrile solution and the crystal by quantitatively estimating the RMSD values (Å).

The root mean square displacement (RMSD)^60^ is suitable for measuring the average distance between the atom-superimposed molecules in different states. Herein, we adopt the RMSD method to measure the average conformational differences for the most stable structures between the S_0_ and S_1_ states in both the solution and the crystal. The RMSD value is estimated as 0.590 (0.930) Å in acetonitrile solution and as 0.294 (0.162) Å in the crystal state between the E and E* forms for TS (TS-OMe), as shown in Fig. 2. The evident structural discrepancies are mainly caused by the rotation of the salicylaldehyde units in the S_1_ states. It is seen that the RMSD value is larger in the solution than in the crystal and this is because the rotation in the crystal is restricted by the steric hindrance and intermolecular interaction arising from the surrounding molecules. In particular, as the TS-OMe molecule is originated from the methoxy substitution of TS, the smaller structural discrepancies between the S_0_ and S_1_ states for the aggregated TS-OMe may due to the stronger interactions between the oxygen atoms and their surrounding molecules.

### 3.1. Interpreting the AIE spectra for the TS and TS-OMe monomers

**Fig. 3:**
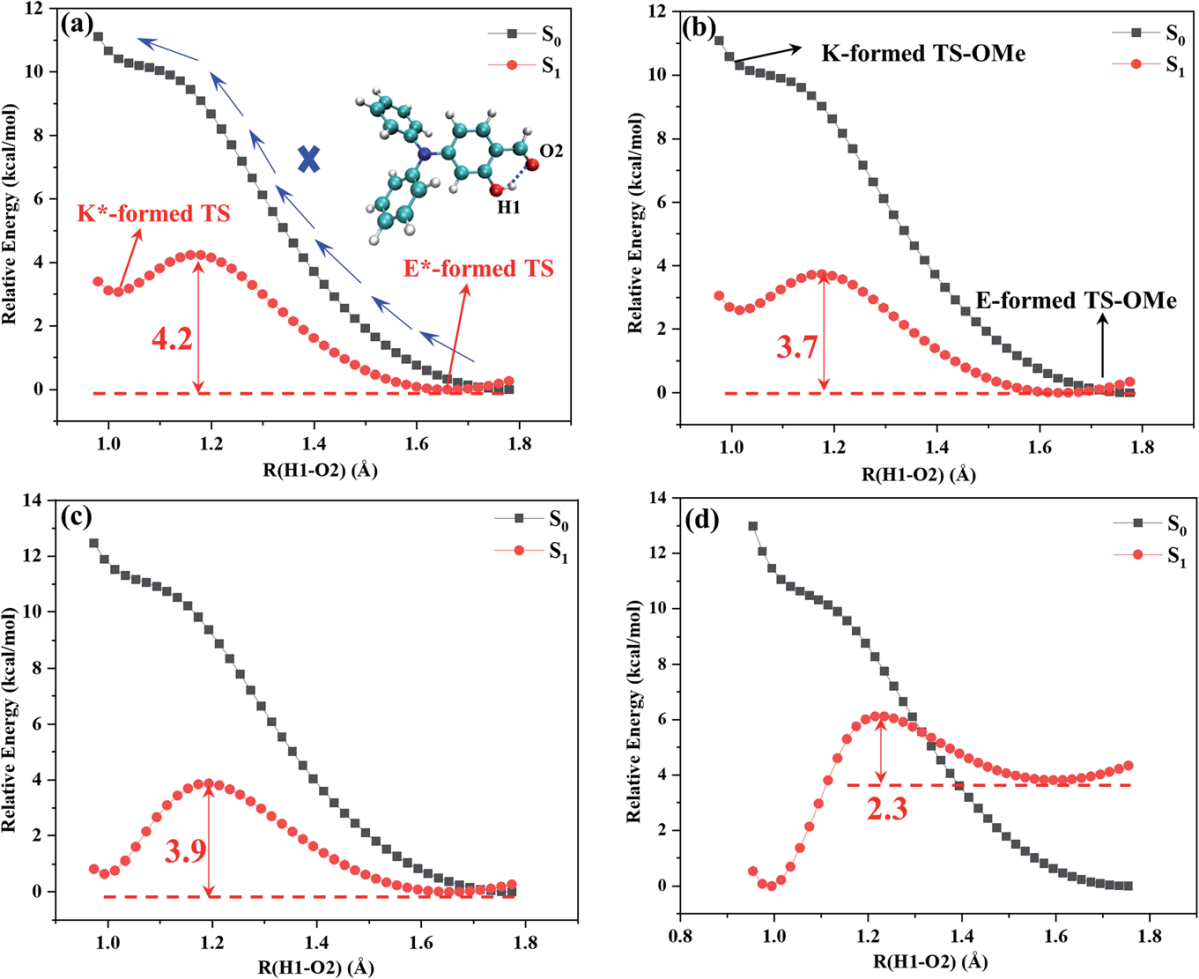
Calculated S_0_ (black) and S_1_ (red) potential energy curves with respect to the H1–O2 bond distance. (a) TS in solution, (b) TS-OMe in solution, (c) TS in crystal, and (d) TS-OMe in crystal.

Potential energy curves were calculated by the partial optimization at a fixed H1–O2 bond distance varying from 0.98 Å to 1.8 Å with a stepsize of 0.02 Å, as shown Fig. 3, in which the energy for the E-form S_0_ (E*-form S_1_ or K*-form S_1_ for TS-OMe) is chosen as the zero point for the ground-state (excited-state) potential energy. Fig. 3 shows that the potential energies of the S_0_ profiles are monotonically increased with respect to the decrease in the H1–O2 distances from the most stable E-form structures to their K-form isomers in both the solution and the crystal for the TS and TS-OMe molecules, and the considerably high energy barriers (more than 10 kcal mol^−1^) further confirm the stability of the E-form isomers in the S_0_ state.

The potential energies in the S_1_ state show that the K*-form energy is 3.1 (2.6) kcal mol^−1^ higher than its corresponding E*-form energy in the solution for TS (TS-OMe), as shown in Fig. 3a and b. Next, we carry out the calculation for the de-excitation energy and its corresponding oscillator strength from the Franck–Condon region at the S_1_ state vertically de-excited to the S_0_ state for the emission spectrum. The calculated vertical de-excitation energy at the E*-form S_1_ state is 422 (518) nm in comparison with experimental observed value of 430 (450) nm for TS (TS-OMe) in solution, and the calculated oscillator strength for emission is 0.422 (0.162), as shown in Table 1. Although it is experimentally stated that the weak emission peaks measured at 430 and 450 nm for TS and TS-OMe in solution are originated from their E* forms, while the experimentally measured emission spectral profiles in solution are abnormally broad, as shown in Fig. S1 (ESI†), it might indicate that the experimental emission spectra are likely obtained from the contribution of both the E* and K* isomers. This fact can be seen from the calculated de-excitation energy at the K*-form S_1_ state, which is 497 (618) nm in solution for TS (TS-OMe) and is red shift by 75 (100) nm with respect to its corresponding E*-form S_1_ state.

**Table 1:** Calculated emission wavelength (*λ*, nm) and oscillator strength (*f*) for the S_1_ → S_0_ transitions for the TS and TS-OMe monomers in both acetonitrile solution and the crystal in comparison with the experimentally measured emission peaks (*λ*_expt._, nm). The energy differences (Δ*E* in eV) between the calculation and the experiment are listed

Structure	E*		K*		*λ*_expt._	Δ*E*
	*λ*	*f*	*λ*	*f*		
In acetonitrile solution						
TS	422	0.422	497	0.142	430	0.05
TS-OMe	518	0.162	618	0.080	450	0.36

In crystal						
TS	380	0.349	454	0.070	493	0.75
TS-OMe	366	0.423	457	0.040	533	0.39

However, in the crystal, the K*-form energy is just 0.6 kcal mol^−1^ higher than its corresponding E*-form energy for TS, as shown in Fig. 3c, in which the K* isomer might be the more favored state by just overcoming 3.8 kcal mol^−1^ potential energy barrier for tautomerization from the E* to the K* form. In contrast, the K*-form energy is 3.5 kcal mol^−1^ lower than its corresponding E*-formed energy for TS-OMe, as shown in Fig. 3d, in which the K* form is the most stable structure in the crystal. As the TS-OMe molecule is originated from the methoxy substitution of TS and the methoxy groups can interact with their surrounding MM molecules, the TS-OMe molecule in the QM region is harder to tautomerize from the E* to the K* form than that of the TS molecules. Therefore, the energy difference between E* and K* is larger for TS-OMe than that for TS. From the thermodynamics point of view, the TS is relatively prone to form the E* isomer in the crystal without the ESIPT process but this might not be true because the calculated emission spectral peak at 380 nm for the E* form has a large discrepancy in comparison with the experimental 493 nm, as shown in Table S6 (ESI†), in which the calculated Stokes' shift of 65 nm is just about half of the experimental value of 127 nm. Therefore, we have a reasonable doubt about the experimental conclusion that the emission spectrum is generated from the excited-state E* monomer for the TS in the crystal. This can be easily seen from Table 1 that the calculated emission spectral peak at 454 nm for the K* form agrees well with the experimental value of 493 nm. Finally, we conclude that the experiment actually observed the emission spectrum of the K* form by experiencing the ESIPT process from the E* to the K* form for TS in the crystal. In the case of the TS-OMe in crystal, it is much clear that the K* form is more stable than its E* form from the thermodynamics point of view. Besides, the calculated emission spectral peak at 457 nm (Stokes' shift 165 nm) for the K* form agrees well with the experimental value of 533 nm (Stokes' shift 166 nm), as shown in Table S6 (ESI†). The present calculation confirms that the experimentally observed emission spectrum is originated from its K* form by experiencing the ESIPT process from the E* to the K* isomer for TS-OMe in the crystal.

We briefly summarize the present simulation results in which the experimentally observed emission spectra are mostly generated from the E* form without the ESIPT process for TS and TS-OMe in solution, while the experimentally observed AIE spectra are generated from the K* form *via* the ESIPT process from the E* to the K* form for both the TS and TS-OMe monomers in the crystal.

### 3.2. Interpreting the AIE spectra for the TS and TS-OMe dimers

**Fig. 4:**
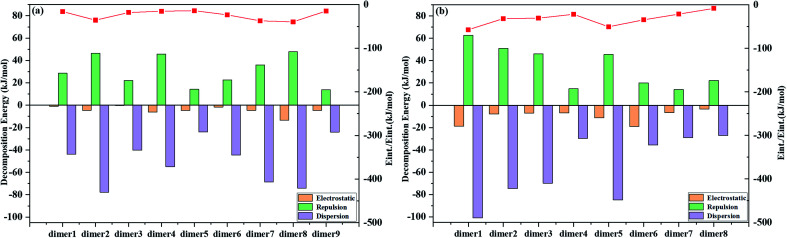
Decomposition of the interaction energies extracted from the ONIOM structures directly adjacent to the QM-region molecule and the red line represents the total interaction energies (*E*_int._). (a) TS dimer and (b) TS-OMe dimer.

We investigate the possible electronic structures of the TS and TS-OMe dimers in the aggregated crystal state by considering that both TS and TS-OMe are loosely piled up with almost no close π⋯π stacking interactions observed in the experimental crystal structures, in which they show highly distorted conformations. The observed short C–H⋯O bonds in both TS and TS-OMe can actually link the adjacent monomers into dimers and form rigidified molecular configurations. Then, we simulate the emission spectra of both the TS and TS-OMe dimers in emission spectra crystal by extracting the surrounding molecules directly adjacent to the central QM-region molecules. Firstly, we preliminarily construct nine and eight representative QM dimers for TS and TS-OMe, respectively. Actually, the intermolecular interactions between the two QM monomers of the selected dimers can be fully analyzed using the classical molecular field (FF) based on the energy decomposition (EDA) approach. The total intermolecular interaction energies can be decomposed into the energies of electrostatic interactions and the van der Waals (vdW) interactions (repulsion and dispersion interactions). The calculated EDA-FF total interaction energies (including electrostatic, repulsive, and dispersion energies) are plotted in Fig. 4 for all the representative dimers of TS and TS-OMe, and the detailed interaction energies of decompositions are summarized in Table S7 (ESI†) as well. The absolute values of the calculated decomposition interaction energies for the TS-OMe dimers are fairly larger than the corresponding energies for the TS dimers. This is because TS-OMe is generated from the methoxy substitution of TS, in which the oxygen atoms generally exhibit considerable electronegativity. Therefore, it is the main reason that stronger and larger intermolecular interaction energies for the TS-OMe dimers may arise from the interactions of the oxygen atoms with their neighbor MM molecules.

As shown in Fig. 4, the dispersion interactions make a primary contribution to the TS and TS-OMe dimerizations, and the electrostatic interactions also make a large contribution to the dimer8 for TS, and dimer1, dimer5, and dimer6 for TS-OMe. Therefore, the combination of dispersion and electrostatic interactions is beneficial for rigidifying the molecular configuration and locking motions; thus, it might inhibit non-radiative relaxation in the aggregated states. As shown in Table S7 (ESI†), the strongest total interaction energies are −39.61 and −57.18 kJ mol^−1^, respectively, for TS dimer8 and TS-OMe dimer1, and the corresponding dimer electronic structures are depicted in Fig. S2 and S3 (ESI†) for TS dimers and TS-OMe dimers, respectively. The TS dimer8 in the crystal is consistent with the experimentally selected most stable dimer configuration, while the TS-OMe dimer1 is not (the actually experimentally selected stable TS-OMe dimer is calculated as the TS-OMe dimer8 with the interaction energy as small as −8.19 kJ mol^−1^ in the crystal, as shown in Table S7 (ESI†)). As there are E-form and K-form monomers for both TS and TS-OMe due to the existence of the strong intramolecular hydrogen bonds, we can classify aggregated TS dimer8 and TS-OMe dimer1 into two E isomers (namely, EE), one E isomer and one K isomer (namely, EK), and two K isomers (namely, KK), and their corresponding electronic configurations are shown in Fig. S4 (ESI†), in which all the surrounding MM molecules are in the E-form. Similar to TS and TS-OMe monomer calculations in which the E forms are proved as the most stable electronic configurations in the S_0_ states, we carry out calculation again in the QM region by the ONIOM-EE((TD)BMK/6-31G(d,p)):UFF method and we found that the EE form are the most stable electronic configurations in the S_0_ state for both the TS-dimer8 and the TS-OMe dimer1. The calculated EE forms are actually consistent with the experimentally observed crystal structures. The ONIOM-EE((TD)BMK/6-31G(d,p)):UFF calculation indicates that the EE* form is the more stable electronic configuration in the excited-state S_1_ for the TS-dimer8, in which the EE* form has energy of 4.2 kcal mol^−1^ lower than the EK*form. However, the same calculation confirms that the EK* form is more stable than the EE* form in the case of the TS-OMe dimer1 (the calculation also confirms that the EK* form is more stable than EE* for the experimentally selected TS-OMe dimer8).

**Fig. 5:**
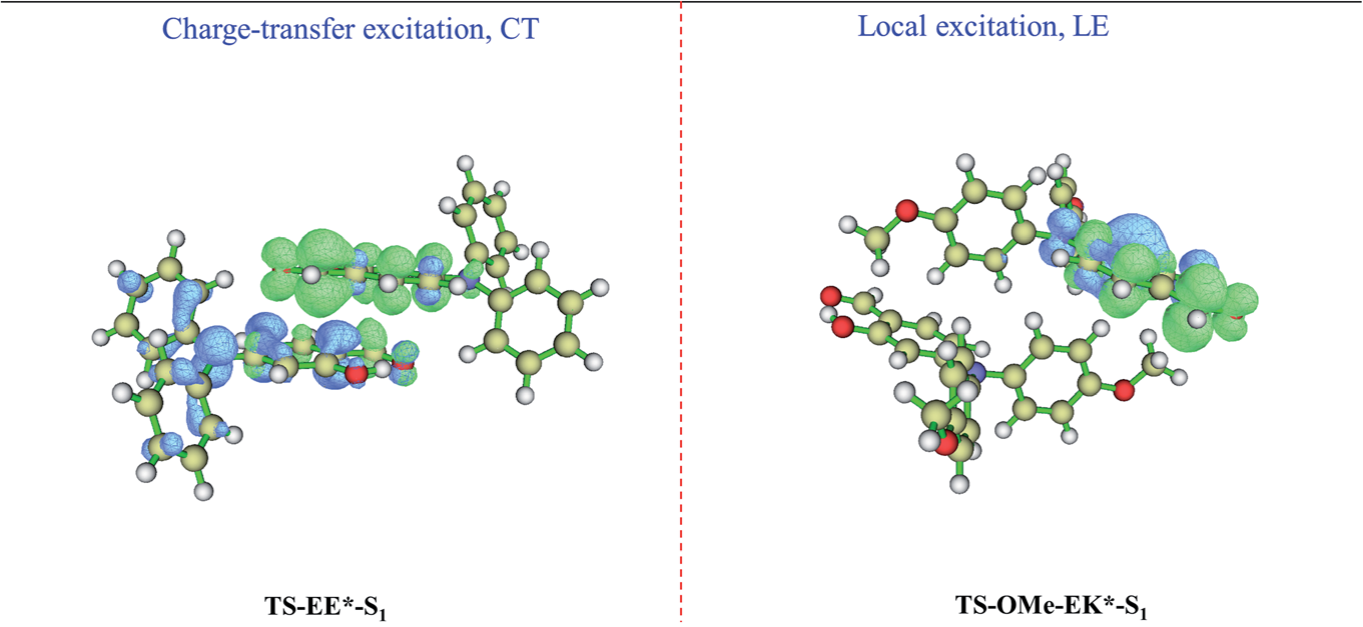
Calculated electron–hole distributions of the EE* (left panel for TS) and EK* (right panel for TS-OMe) dimers in the S_1_ state, where the blue and green isosurfaces represent the hole and electron distributions, respectively.

We perform absorption (emission) spectrum calculation by estimating the vertical excitation (de-excitation) energy from the Franck–Condon region at EE on the S_0_ state (at EE* on S_1_ state) for the TS-dimer8, and the calculated absorption of 337 nm and emission 456 nm are in good agreement with the experimentally observed absorption of 366 nm and emission of 493 nm, as shown in Table 2. In particular, the calculated Stokes' shift of 119 nm agrees well with the experimental value of 127 nm. It is interesting that the emission of 456 nm at EE* for the TS-dimer8 is consistent with the emission of 454 nm at K* (not 380 nm at E*) for the TS monomer in the crystal. The simulated results also show the accuracy of the selected models and the methods. The present analysis concludes that although the observed emission spectrum for the TS monomer in the crystal is generated from the K* form *via* the ESIPT process from the E* to the K* form, the EE* form of the TS dimer may also contribute to the AIE mechanisms in the aggregated states. Furthermore, we carry out calculations for the electron–hole distributions for the EE* TS-dimer8 on the S_1_ state, in which a charge-transfer excitation from one monomer to another takes place, as shown in the left panel of Fig. 5. Besides, the distance for the π⋯π interactions between the two E-form monomers in EE* is smaller than the corresponding distance in the EE on its ground state, as shown in Fig. S5 (ESI†), which indicates that there is stronger π⋯π interactions in the excited state S_1_. This evidence reinforces the idea that the EE* dimers also contribute to the AIE mechanisms in the aggregated states.

**Table 2:** Calculated absorption and emission wavelengths (*λ*, nm) from the TS (EE*) and TS-OMe (EK*) dimers in the crystal in comparison with the experimental values. The corresponding Stokes' shifts (nm) in both the calculation and the experiment are compared

	Cal.				Expt.		
	*λ*_abs_	*λ*_emi_	*f*	Stokes'	*λ*_abs_	*λ*_emi_	Stokes'
TS	337	456	0.029	119	366	493	127
TS-OMe	292	459	0.032	167	367	533	166

On the other hand, the emission spectrum generated from the EK* form on the S_1_ state for the TS-OMe dimer in the crystal involves the ESIPT process. The calculated absorption of 292 nm, emission of 459 nm, and its corresponding Stokes' shift of 167 nm are in reasonably good agreement with the experimentally observed absorption of 367 nm, emission of 533 nm, and Stokes' shift of 166 nm, as shown in Table 2 for the TS-OMe dimer in the crystal. In particular, the calculated Stokes' shift of 167 nm agrees perfectly well with the experimental value of 166 nm. Besides, the calculated emission of 459 nm from EK* in the TS-OMe dimer is almost the same as the calculated emission of 457 nm from K* in the TS-OMe monomer; thus, the observed emission spectra for both the dimer and monomer involves the ESIPT process for TS-OMe in the crystal. Actually, the analysis from electron–hole distribution clearly shows that there is no charge-transfer excitation from one monomer to another but all the charge is localized in the single K* monomer in the EK* state, as shown in the right panel of Fig. 5. Therefore, the dimers exhibit a negligible influence on the emission but the K* monomer is responsible for the AIE emission of the TS-OMe dimer in the crystal *via* the ESIPT process.

We briefly summarize the present simulation results in which the experimentally observed AIE spectra are generated from the EE* form *via* intermolecular charge-transfer excitation instead of the ESIPT process for the TS dimer in the aggregated crystal state, while the dimers for TS-OMe exhibit a negligible influence on the emission for the experimentally observed AIE spectra.

### 3.3. Nonadiabatic dynamics simulation for the TS and TS-OMe monomers

**Fig. 6:**
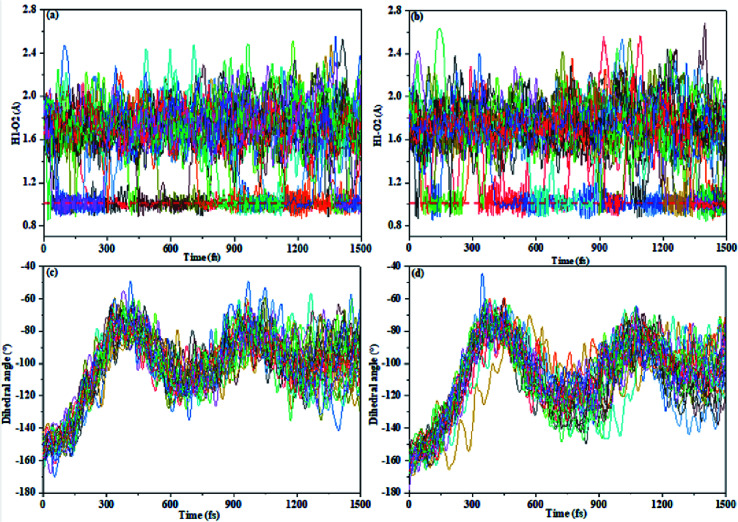
The bond length H1–O2 and dihedral angle (*θ*) varies with respect to time for total 27 sampling trajectories (the ESIPT process is determined by the H1–O2 distance around 1.0 Å). (a) H1–O2 in TS, (b) H1–O2 in TS-OMe, (c) *θ* in TS, and (d) *θ* in TS-OMe.

We present certain qualitative discussions from another aspect for the AIE spectra by performing trajectory surface hopping molecular dynamics simulation on on-the-fly TD-BMK/6-31G(d) potential energy surfaces up to excitation to the S_1_ state from the E-form Franck–Condon region on the S_0_ state, and we treat the TS and TS-OMe monomers to be in the gas phase for simplicity. We found no hopping event for total 27 sampling trajectories for both the TS and TS-OMe monomers up to 1500 fs evolution. However, we can see the ESIPT process that takes place from Fig. 6 (as the H1–O2 bond distance reaches 1.0 (1.8) Å in average, it means that trajectories trap on the K* (E*) form on the S_1_ state). We can see from the H1–O2 bond evolution features shown in Fig. 6a and b that about half of the sampling trajectories are on K* and the rest are on E* for the TS (TS-OMe) monomers. This nonadiabatic dynamics simulation indicates that all the trapped trajectories undergo fluorescence emission from either the K* or the E* form but the experimental measurement showed very weak emission in solution for both TS and TS-OMe. This must be due to the strong interaction between the solvent molecules and the solute molecule so that the energy quickly dissipates into the solution before the possible emission process could take place. Fig. 6c and d also shows that the dihedral angle *θ* (between the triphenylamine and salicylaldehyde planes) rotates in a similar pattern during the evolution for all the sampling trajectories for TS (TS-OMe), and its average rotation *θ* agrees well with the rotation of TS and TS-OMe from the S_0_ to the S_1_ states, as shown in Table S5 (ESI†). On the other hand, if we could perform trajectory surface hopping molecular dynamics simulation in the aggregated crystal states, this rotation *θ* motion must be significantly restricted, as shown in Table S1 (ESI†). Besides, the interaction between the surrounding MM molecules and the QM molecule must be significantly weakened in the aggregated crystal states. This implicitly indicates that the AIE spectra can be enhanced in the aggregated crystal states for both TS and TS-OMe as analyzed in the previous section from the molecular spectral simulation.

## 4. Conclusions

We have performed (TD)BMK/6-31G(d,p) + PCM calculation in acetonitrile solution and ONIOM-EE((TD)BMK/6-31G(d,p)):UFF calculation in the aggregated crystal state for both the TS and TS-OMe molecules. Molecular spectral simulation indicates that the AIE spectra in the crystal undergo the ESIPT process from the E* to the K* form for both the TS and TS-OMe monomers, while the simulated emission spectra in acetonitrile solution for both the TS and the TS-OMe monomers are driven by the E* form without the ESIPT process. All the simulations agree with the experimental results. Furthermore, molecular spectral simulation has been carried out for the TS and TS-OMe dimers in the crystal. The calculated absorption and emission peaks agree well with the experimental values, especially for the obtained Stokes' shifts. Besides, the EE* dimers of TS also contribute to emission in the crystal with the charge transfer from one monomer to another. Conversely, the EK* dimers have a negligible influence on the AIE spectra mechanism for TS-OMe in the crystal with the charge being localized in the K* monomer. The present molecular spectral simulations have well interpreted the AIE + ESIPT mechanisms observed in the experiment for both the TS and TS-OMe molecules in aggregated crystal states. The observations that emission spectra are weak in solution and enhanced in the aggregated states are briefly explained by performing nonadiabatic molecular dynamics simulation for both the TS and TS-OMe monomers.

The present molecular spectral and dynamics simulations present a deep understanding of the AIE photophysical mechanisms combined with the ESIPT process for triphenylamine salicylaldehyde derivatives (monomers and dimers), which can provide physical insights into the design of highly efficient AIE compounds.

## Conflicts of interest

The authors declare no competing financial interests.

## Supplementary Material

RA-011-D1RA07388E-s001
