# Supplementary material for: Aggregation-induced emission spectra of triphenylamine salicylaldehyde derivatives via excited-state intramolecular proton transfer revealed by molecular spectral and dynamics simulations
Source: RSC Adv. 2021 Nov 18;11(59):37171–80. doi: 10.1039/d1ra07388e (PMC9043594; doi:10.1039/d1ra07388e)
Supplement: RA-011-D1RA07388E-s001 [file RA-011-D1RA07388E-s001.pdf]

Electronic Supplementary Material (ESI) for RSC Advances.
This journal is © The Royal Society of Chemistry 2021

Support information for

Aggregation-induced emission spectra of triphenylamine salicylaldehyde derivatives via excited-state intramolecular proton transfer revealed by molecular spectral and dynamic simulations

Qing Zhang,[a] Yuanyuan Li,[a] Zexing Cao*[a] and Chaoyuan Zhu*[cb]

[a]*State Key Laboratory of Physical Chemistry of Solid Surfaces and Fujian Provincial Key Laboratory of Theoretical and Computational Chemistry, College of Chemistry and Chemical Engineering, Xiamen University, Xiamen 360015, China.*

[b]*Department of Applied Chemistry and Institute of Molecular Science, National Chiao-Tung University, Hsinchu 30010, Taiwan.*

[c]*Department of Applied Chemistry and Center for Emergent Functional Matter Science, National Yang Ming Chiao Tung University, Hsinchu 30010, Taiwan.*

Corresponding Authors:

***E-mails:** zxcao@xmu.edu.cn and cyzhu@mail.nctu.edu.tw**

Table S1. Cartesian coordinates of QM region atoms for S_0 (enol and keto forms) states for TS in its ONIOM model, simulated by using the ONIOM-EE(BMK/6-31G(d,p):UFF approach.

enol				keto			
N	0.28924700	0.51469500	-0.55735900	N	20.11400000	14.22000000	12.23000000
O	1.26095400	5.08248300	-1.55798500	O	21.82700000	9.83400000	11.51400000
O	0.33797700	6.93390600	-0.54209700	O	23.62800000	8.93800000	13.22100000
C	0.79197000	2.83934300	-1.04603800	C	20.98000000	11.99300000	11.88000000
C	0.49015800	4.19879600	-0.92385500	C	21.81500000	10.95600000	12.25800000
C	0.01263600	1.86952000	-0.41775800	C	20.95100000	13.17700000	12.60900000
C	0.70294600	-0.46548600	-0.25912300	C	20.40400000	15.56200000	12.64200000
C	1.13270800	2.27875500	0.36085600	C	21.78000000	13.31100000	13.73800000
C	1.63625500	0.06221300	-0.66643500	C	18.86600000	13.98000000	11.58200000
C	0.50518200	-1.37027300	0.79082000	C	19.48500000	16.28000000	13.38100000
C	0.63368100	4.61291400	-0.14925300	C	22.64700000	11.08300000	13.38300000
C	1.42211000	3.62557800	0.47579900	C	22.60800000	12.28400000	14.09200000
C	0.96856000	6.02127900	-0.01698700	C	23.53500000	10.02200000	13.77900000
C	2.88811500	-1.43575400	-0.67426600	C	21.89600000	17.43000000	12.72700000
C	1.89226600	-0.51094800	-0.99925400	C	21.60900000	16.14400000	12.30000000
C	1.95735300	-0.98611200	-1.54224300	C	18.45400000	14.77700000	10.54000000
C	1.49808300	-2.30110100	1.10028400	C	19.78600000	17.56500000	13.78600000
C	2.69858700	-2.33578500	0.38177300	C	20.99300000	18.14000000	13.46900000
C	2.63220300	0.61484900	0.15218000	C	18.02400000	12.98300000	12.03800000
C	4.26274400	-0.91921500	-0.77249000	C	16.37800000	13.57400000	10.42200000
C	3.26613000	-1.48031400	-1.58100700	C	17.20400000	14.56700000	9.96800000
C	3.94288000	0.13660200	0.08843200	C	16.78900000	12.78300000	11.45600000
H	1.64622300	2.54729600	-1.64542600	H	20.45900000	11.90000000	11.11300000
H	2.27673300	3.94821700	1.06798200	H	23.21500000	12.35000000	14.88700000
H	0.41959000	-1.33095800	1.35858100	H	18.64500000	15.87600000	13.61300000
H	1.73441400	1.53612800	0.87151000	H	21.77100000	14.09800000	14.23600000
H	2.02649300	0.19053300	-1.81349900	H	22.22600000	15.60200000	11.77700000
H	3.46985800	-3.05540900	0.64290600	H	21.19400000	19.12200000	13.79000000
H	2.34912000	1.41140300	0.83120300	H	18.32000000	12.44700000	12.82300000
H	3.80423700	-1.45542600	-1.25881700	H	22.71900000	17.82000000	12.46200000
H	1.17564500	-1.40899100	-2.16743700	H	19.01600000	15.45800000	10.27600000
H	1.85947700	6.25183200	0.60249700	H	24.09300000	10.23300000	14.60000000
H	1.33085700	-2.98970600	1.91787700	H	19.11800000	18.04500000	14.25900000
H	5.27299900	-1.31424500	-0.80129100	H	15.41400000	13.38800000	10.05000000
H	3.51561000	-2.31151300	-2.23458400	H	16.99400000	15.12000000	9.33500000
H	4.70865700	0.56865100	0.72827500	H	16.22600000	12.06800000	11.76700000
H	0.88993800	5.97527000	-1.38922100	H	22.97029472	9.14611154	12.43951992

Table S2. Cartesian coordinates of QM region atoms for S_1 (enol* and keto* forms) states for TS in its ONIOM model, simulated by using the ONIOM-EE((TD)BMK/6-31G(d,p):UFF approach.

enol*				keto*			
N	0.28924700	0.51469500	-0.55735900	N	0.28924700	0.51469500	-0.55735900
O	1.26095400	5.08248300	-1.55798500	O	1.26095400	5.08248300	-1.55798500
O	-0.33797700	6.93390600	-0.54209700	O	-0.33797700	6.93390600	-0.54209700
C	0.79197000	2.83934300	-1.04603800	C	0.79197000	2.83934300	-1.04603800
C	0.49015800	4.19879600	-0.92385500	C	0.49015800	4.19879600	-0.92385500
C	-0.01263600	1.86952000	-0.41775800	C	-0.01263600	1.86952000	-0.41775800
C	-0.70294600	-0.46548600	-0.25912300	C	-0.70294600	-0.46548600	-0.25912300
C	-1.13270800	2.27875500	0.36085600	C	-1.13270800	2.27875500	0.36085600
C	1.63625500	0.06221300	-0.66643500	C	1.63625500	0.06221300	-0.66643500
C	-0.50518200	-1.37027300	0.79082000	C	-0.50518200	-1.37027300	0.79082000
C	-0.63368100	4.61291400	-0.14925300	C	-0.63368100	4.61291400	-0.14925300
C	-1.42211000	3.62557800	0.47579900	C	-1.42211000	3.62557800	0.47579900
C	-0.96856000	6.02127900	-0.01698700	C	-0.96856000	6.02127900	-0.01698700
C	-2.88811500	-1.43575400	-0.67426600	C	-2.88811500	-1.43575400	-0.67426600
C	-1.89226600	-0.51094800	-0.99925400	C	-1.89226600	-0.51094800	-0.99925400
C	1.95735300	-0.98611200	-1.54224300	C	1.95735300	-0.98611200	-1.54224300
C	-1.49808300	-2.30110100	1.10028400	C	-1.49808300	-2.30110100	1.10028400
C	-2.69858700	-2.33578500	0.38177300	C	-2.69858700	-2.33578500	0.38177300
C	2.63220300	0.61484900	0.15218000	C	2.63220300	0.61484900	0.15218000
C	4.26274400	-0.91921500	-0.77249000	C	4.26274400	-0.91921500	-0.77249000
C	3.26613000	-1.48031400	-1.58100700	C	3.26613000	-1.48031400	-1.58100700
C	3.94288000	0.13660200	0.08843200	C	3.94288000	0.13660200	0.08843200
H	1.64622300	2.54729600	-1.64542600	H	1.64622300	2.54729600	-1.64542600
H	-2.27673300	3.94821700	1.06798200	H	-2.27673300	3.94821700	1.06798200
H	0.41959000	-1.33095800	1.35858100	H	0.41959000	-1.33095800	1.35858100
H	-1.73441400	1.53612800	0.87151000	H	-1.73441400	1.53612800	0.87151000
H	-2.02649300	0.19053300	-1.81349900	H	-2.02649300	0.19053300	-1.81349900
H	-3.46985800	-3.05540900	0.64290600	H	-3.46985800	-3.05540900	0.64290600
H	2.34912000	1.41140300	0.83120300	H	2.34912000	1.41140300	0.83120300
H	-3.80423700	-1.45542600	-1.25881700	H	-3.80423700	-1.45542600	-1.25881700
H	1.17564500	-1.40899100	-2.16743700	H	1.17564500	-1.40899100	-2.16743700
H	-1.85947700	6.25183200	0.60249700	H	-1.85947700	6.25183200	0.60249700
H	-1.33085700	-2.98970600	1.91787700	H	-1.33085700	-2.98970600	1.91787700
H	5.27299900	-1.31424500	-0.80129100	H	5.27299900	-1.31424500	-0.80129100
H	3.51561000	-2.31151300	-2.23458400	H	3.51561000	-2.31151300	-2.23458400
H	4.70865700	0.56865100	0.72827500	H	4.70865700	0.56865100	0.72827500
H	0.88993800	5.97527000	-1.38922100	H	0.36044827	6.38864382	-1.02393231

Table S3. Cartesian coordinates of QM region atoms for S_0 (enol and keto forms) states for TS-OMe in its ONIOM model, simulated by using the ONIOM-EE(BMK/6-31G(d,p):UFF approach.

enol				keto			
N	-0.05415700	0.11222600	-0.33538800	N	-0.04993800	0.10272700	-0.33757400
O	-0.03922300	5.64523600	0.36610800	O	-0.04370500	5.63533500	0.36632000
C	-1.09347100	-2.05800500	-0.53882800	C	-1.08462400	-2.06914300	-0.54305200
H	-0.13509300	-2.55100400	-0.48023700	H	-0.12513500	-2.55932100	-0.48449200
C	-2.47879900	-0.03298200	-0.51511600	C	-2.47443100	-0.04716800	-0.51969000
H	-2.56535000	1.04461100	-0.43206200	H	-2.56335000	1.03024800	-0.43666200
C	1.25401200	-0.46885900	-0.27841500	C	1.25986100	-0.47510200	-0.27860900
O	-2.08038500	-4.16518800	-0.81057800	O	-2.06685500	-4.17869500	-0.81501700
H	-2.97286400	-4.55133200	-0.94576100	H	-2.95836700	-4.56608200	-0.95206700
C	-1.18835200	-0.65136800	-0.45764700	C	-1.18241200	-0.66280900	-0.46117800
C	-0.11006300	1.53496400	-0.18829300	C	-0.10853200	1.52538600	-0.19012700
C	-0.06199500	4.32418400	0.11750000	C	-0.06445300	4.31436600	0.11709400
C	-0.37548500	2.11190900	1.06251800	C	-0.37558700	2.10132000	1.06081400
H	-0.60405200	1.46529800	1.90428300	H	-0.60380700	1.45372600	1.90192400
O	5.08016200	-2.05250800	0.03186900	O	5.09223500	-2.04254200	0.03687400
C	0.16207400	2.35750500	-1.28186800	C	0.16353300	2.34867300	-1.28309800
H	0.37782100	1.89407800	-2.24061100	H	0.38114400	1.88599100	-2.24180100
C	-0.33763700	3.49195900	1.22069400	C	-0.33963500	3.48133300	1.21982700
H	-0.50184400	3.94144900	2.19197400	H	-0.50484800	3.93042900	2.19118700
C	0.17653100	3.75062200	-1.14299400	C	0.17576800	3.74173900	-1.14352200
H	0.39227700	4.37560400	-1.99950100	H	0.39133900	4.36740900	-1.99957800
C	1.84470100	-1.02852900	-1.41509500	C	1.85484000	-1.03129400	-1.41472300
H	1.28396600	-1.04926700	-2.34746600	H	1.29610600	-1.05346400	-2.34822300
C	3.13264700	-1.58104300	-1.35705300	C	3.14510000	-1.57828100	-1.35491400
H	3.56934100	-2.01274100	-2.25188500	H	3.58537400	-2.00683300	-2.24951000
C	-3.51531800	-2.22928600	-0.81554300	C	-3.50594300	-2.24588100	-0.82095300
C	-3.59683600	-0.82129700	-0.69775600	C	-3.59057100	-0.83768000	-0.70350100
H	-4.57952500	-0.35519900	-0.75645900	H	-4.57405300	-0.37318100	-0.76362200
C	3.24228400	-0.99782000	1.00594700	C	3.24797200	-0.99713800	1.00887500
H	3.80887500	-0.99094300	1.93325800	H	3.81303400	-0.98839300	1.93710100
C	-2.22963200	-2.84196300	-0.71794000	C	-2.21871800	-2.85563400	-0.72268100
C	3.83865100	-1.56336900	-0.13976800	C	3.84876800	-1.55895500	-0.13638500
C	1.96381200	-0.45586400	0.93360500	C	1.96744100	-0.46037900	0.93473100
H	1.50373200	-0.00384500	1.80838800	H	1.50420100	-0.01069700	1.80903100
C	0.29007700	6.53103400	-0.68415100	C	0.28252600	6.52233900	-0.68387100
H	0.34463600	7.52330300	-0.23043300	H	0.33560100	7.51454700	-0.22982600
H	-0.48053200	6.52732800	-1.46851800	H	-0.48905900	6.51737200	-1.46727000
H	1.26736500	6.28953200	-1.12388200	H	1.25981100	6.28310000	-1.12477300
O	-4.68629100	-4.25747400	-1.18263200	O	-4.67317500	-4.27700800	-1.18623800
C	5.73889200	-2.63685800	-1.07346800	C	5.75643400	-2.62126000	-1.06812500
H	5.89206800	-1.90760000	-1.88160900	H	5.91189300	-1.88806000	-1.87230500
H	5.17686000	-3.49408100	-1.47027700	H	5.19768800	-3.47760800	-1.47127500
H	6.70677400	-2.97953700	-0.69999100	H	6.72308800	-2.96431400	-0.69188000
C	-4.69036900	-3.03050800	-1.06929400	C	-4.67884100	-3.04953500	-1.07403600
H	-5.64101600	-2.46926600	-1.17555700	H	-5.63047600	-2.49024600	-1.18112600

Table S4. Cartesian coordinates of QM region atoms for S_1 (enol* and keto* forms) states for TS-OMe in its ONIOM model, simulated by using the ONIOM-EE((TD)BMK/6-31G(d,p):UFF approach.

enol*				keto*			
N	-0.06885500	0.10860400	-0.24392600	N	-0.04325000	0.13443200	-0.26714200
O	-0.02806800	5.62633100	0.30588400	O	-0.02823900	5.66800300	0.33441700
C	-1.14211700	-2.08042200	-0.37761200	C	-1.08536900	-2.03689700	-0.42227000
H	-0.20655100	-2.56022300	-0.12219600	H	-0.14581200	-2.51120900	-0.17136000
C	-2.50926500	-0.06194500	-0.63285900	C	-2.46344400	-0.01893200	-0.55684300
H	-2.59949700	1.02064600	-0.64956800	H	-2.59567200	1.05576700	-0.51957900
C	1.20293700	-0.44122800	-0.31979600	C	1.25513700	-0.44765900	-0.25906300
O	-2.10119900	-4.21339800	-0.63076300	O	-2.03752600	-4.16147800	-0.69498800
H	-3.01682100	-4.58135900	-0.82254500	H	-3.63870900	-4.66135400	-0.99239300
C	-1.26406000	-0.66601400	-0.42354600	C	-1.19718200	-0.59313900	-0.42246400
C	-0.10083100	1.50903200	-0.10090800	C	-0.08823200	1.55662200	-0.14224800
C	-0.02931900	4.30771500	0.09865700	C	-0.04286600	4.34581400	0.10774400
C	-0.56100600	2.14012100	1.07237200	C	-0.42457600	2.15422600	1.08108200
H	-0.93347000	1.53287300	1.89211700	H	-0.70463800	1.52589000	1.92102100
O	4.99639900	-2.10086200	-0.13112400	O	5.06359700	-2.06666500	-0.01634000
C	0.37958900	2.29376500	-1.16987500	C	0.25140100	2.35319700	-1.23880500
H	0.71714400	1.78849400	-2.07156500	H	0.51429200	1.87019800	-2.17641500
C	-0.46188000	3.51552500	1.18945600	C	-0.37079800	3.53503400	1.21374400
H	-0.68698900	4.01214200	2.12093600	H	-0.56100900	4.00516000	2.16895100
C	0.37389200	3.68575900	-1.09877900	C	0.25360800	3.74791800	-1.12987800
H	0.70260800	4.27594000	-1.94427200	H	0.50830800	4.35580100	-1.98788700
C	1.63996700	-1.27013700	-1.38733600	C	1.76586000	-1.13098600	-1.37179800
H	0.96682900	-1.45352800	-2.22117600	H	1.15358800	-1.22226900	-2.26686600
C	2.92285800	-1.81371400	-1.37835700	C	3.05140600	-1.68952100	-1.33686800
H	3.25817300	-2.40635500	-2.22222100	H	3.43463300	-2.20143600	-2.21333600
C	-3.53612400	-2.29585800	-0.87784300	C	-3.50475800	-2.27896200	-0.86235800
C	-3.63648100	-0.87198300	-0.82945900	C	-3.57226600	-0.87682900	-0.77373400
H	-4.61321000	-0.41688600	-0.97340900	H	-4.55069000	-0.41157400	-0.89513100
C	3.31672800	-0.81960700	0.83117000	C	3.30061600	-0.90171800	0.96020700
H	3.97497800	-0.70580800	1.68893100	H	3.91584100	-0.83557200	1.85383900
C	-2.24884600	-2.88907600	-0.63214400	C	-2.20421300	-2.91239100	-0.66982600
C	3.76760100	-1.60748100	-0.26111200	C	3.82428600	-1.57833400	-0.16570900
C	2.08641600	-0.20419900	0.77653200	C	2.04167000	-0.32450100	0.90415600
H	1.73333900	0.40818100	1.60258500	H	1.63866200	0.21469500	1.75786400
C	0.34884800	6.49734800	-0.74682400	C	0.32064200	6.54134100	-0.72145600
H	0.34571700	7.50013900	-0.31569700	H	0.35587400	7.53977000	-0.28062800
H	-0.37218000	6.44820500	-1.57425700	H	-0.43132100	6.51892300	-1.52298000
H	1.35770300	6.27136900	-1.11462800	H	1.31024500	6.30125300	-1.13277500
O	-4.56770100	-4.42785200	-1.17459000	O	-4.58306100	-4.42524700	-1.17237000
C	5.54537300	-2.89580000	-1.17029700	C	5.66776900	-2.74326500	-1.10283700
H	5.59692800	-2.33367900	-2.11075300	H	5.76992000	-2.08431700	-1.97563800
H	4.95054800	-3.80637500	-1.32263800	H	5.08965800	-3.63238400	-1.39071300
H	6.55129600	-3.16005500	-0.83855300	H	6.65661100	-3.04762000	-0.75265700
C	-4.64705700	-3.16609200	-1.16939000	C	-4.63463300	-3.09130900	-1.12615500
H	-5.60635200	-2.67414000	-1.41070800	H	-5.61612100	-2.66067100	-1.30461200

Table S5. Key Geometry parameters calculated by (TD)BMK/6-31G(d,p)+PCM method in acetonitrile solution and by ONIOM-EE((TD)-BMK/6-31G(d,p):UFF) method in aggregated crystal for both TS and TS-OMe. Dihedral angles are in degrees (θ°) and bond lengths are in Angstrom (Å), and relative energies are in kcal·mol^{-1}. The 0.0 kcal·mol^{-1} (ΔE) is chosen as zero point of energy for the lower minimum in either S_0 or S_1 states, separately.

structure		geometric parameters			ΔE (opt)
		θ	R(H1-O1)	R(H1-O2)	
in solution					
TS	E	154.90	0.98	1.78	0.0
	E*	121.36	0.99	1.66	0.0
	K*	105.50	1.57	1.01	3.1
TS-OMe	E	-160.21	0.98	1.78	0.0
	E*	-103.22	1.00	1.64	0.0
	K*	-98.77	1.55	1.02	2.6
in crystal					
TS	E	-162.24	0.98	1.77	0.0
	E*	-142.36	1.00	1.66	0.0
	K*	-152.74	1.69	0.99	2.6
TS-OMe	E	-177.07	0.98	1.75	0.0
	E*	-171.72	1.01	1.60	1.7
	K*	-174.59	1.70	0.99	0.0

Table S6. Calculated absorption and emission wavelength (λ, nm) for the TS and TS-OMe monomers and dimers in crystal in comparison with experimentally measured wavelength. The corresponding Stokes shifts (nm) in both calculation and experiment are compared.

structure		**Cal.**			**Expt**		
		absorption	emission	Stokes	absorption	emission	Stokes
TS	monomer	315	380	65	366	493	127
	dimer	337	456	119			
TS-OMe	monomer	292	457	165	367	533	166
	dimer	292	459	167			

Table S7. Decomposition interaction energies calculated by the EDA-FF approach for the representative dimers of TS and TS-OMe in crystal. The energy unit is kJ/mol.

TS	Elec.	Rep.	Disp.	Total	TS-OMe	Elec.	Rep.	Disp.	Total
dimer1	-0.96	28.63	-43.76	-16.09	dimer1	-18.73	62.55	-100.99	-57.18
dimer2	-4.51	46.41	-77.74	-35.83	dimer2	-7.85	50.82	-74.64	-31.67
dimer3	-0.15	22.16	-40.28	-18.27	dimer3	-6.95	46.05	-69.82	-30.72
dimer4	-6.03	45.76	-54.96	-15.23	dimer4	-6.86	14.76	-29.60	-21.69
dimer5	-4.63	14.40	-23.77	-14.00	dimer5	-11.22	45.57	-84.87	-50.51
dimer6	-1.72	22.68	-44.62	-23.65	dimer6	-19.00	20.02	-35.56	-34.55
dimer7	-4.57	35.92	-68.61	-37.26	dimer7	-6.65	14.13	-28.90	-21.42
dimer8	-13.4	47.84	-74.06	-39.61	dimer8	-3.43	22.26	-27.02	-8.19
dimer9	-4.73	13.83	-23.99	-14.89					

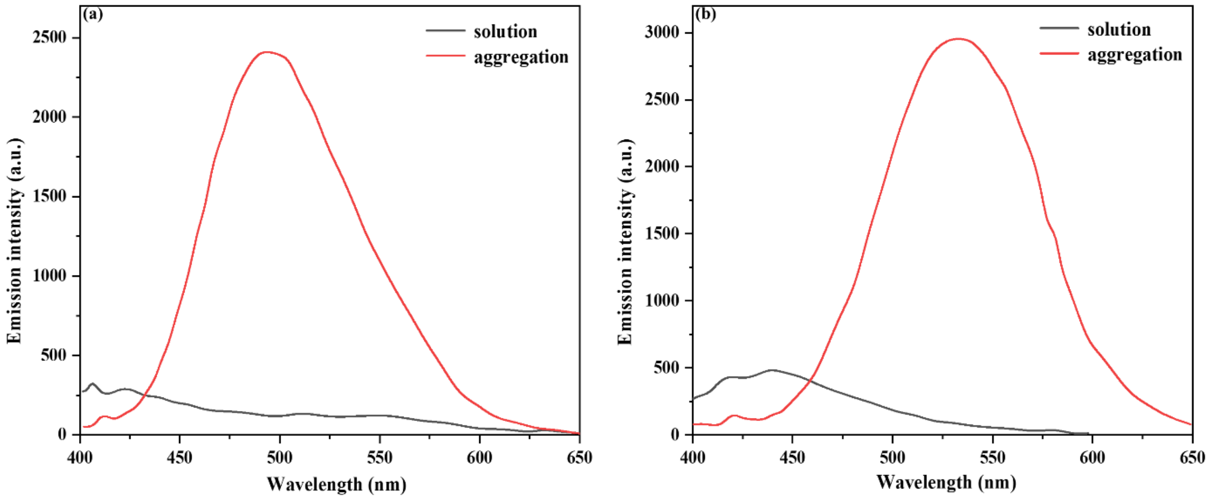

Fig. S1 The experimentally measured emission spectra in acetonitrile/water mixtures with f_w of 0% (in solution) and 99% (in aggregation), respectively for TS (left panel) and TS-OMe (right panel).[23]

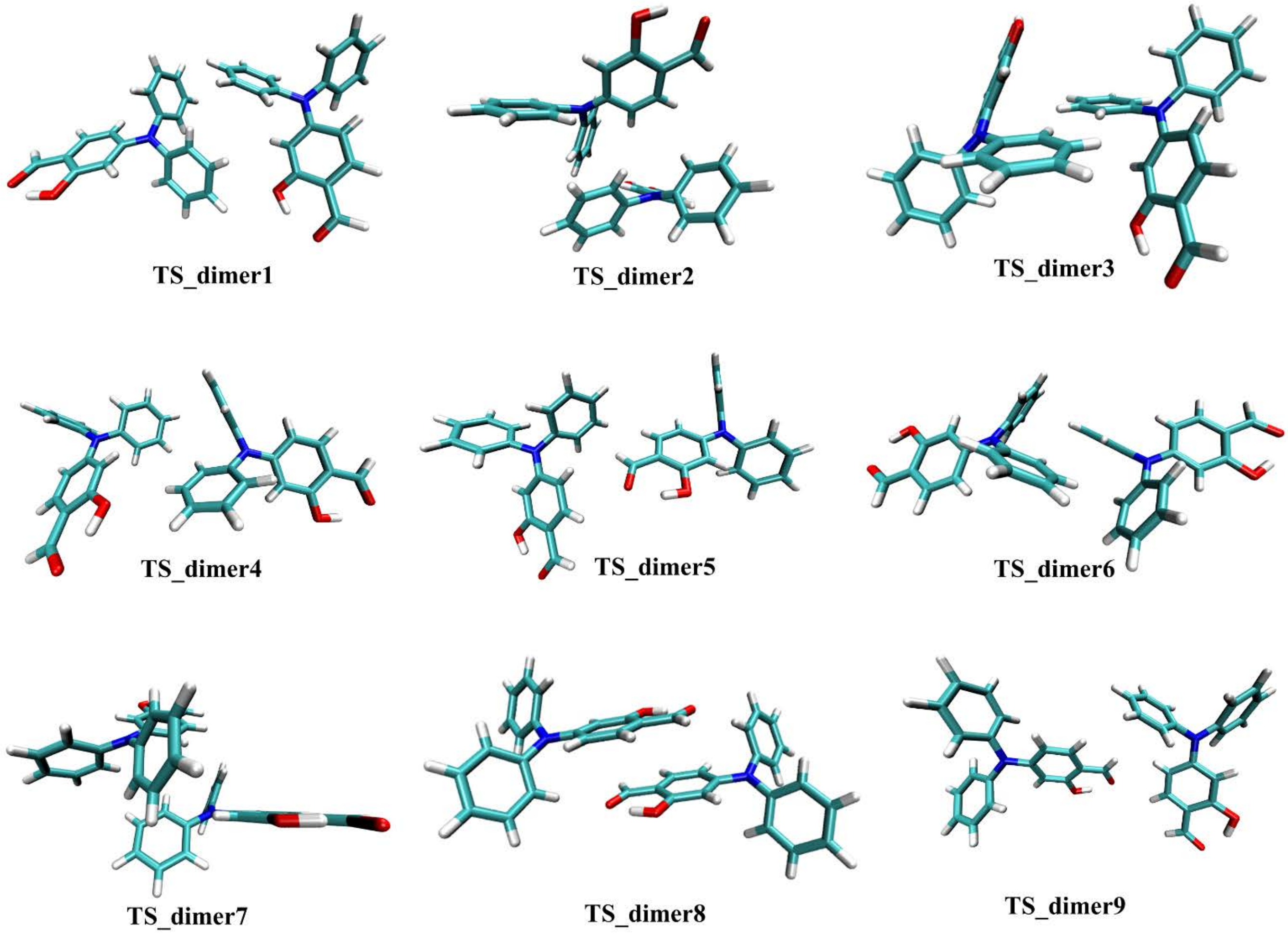

Fig. S2 The geometries of all the representative dimers of TS in crystal.

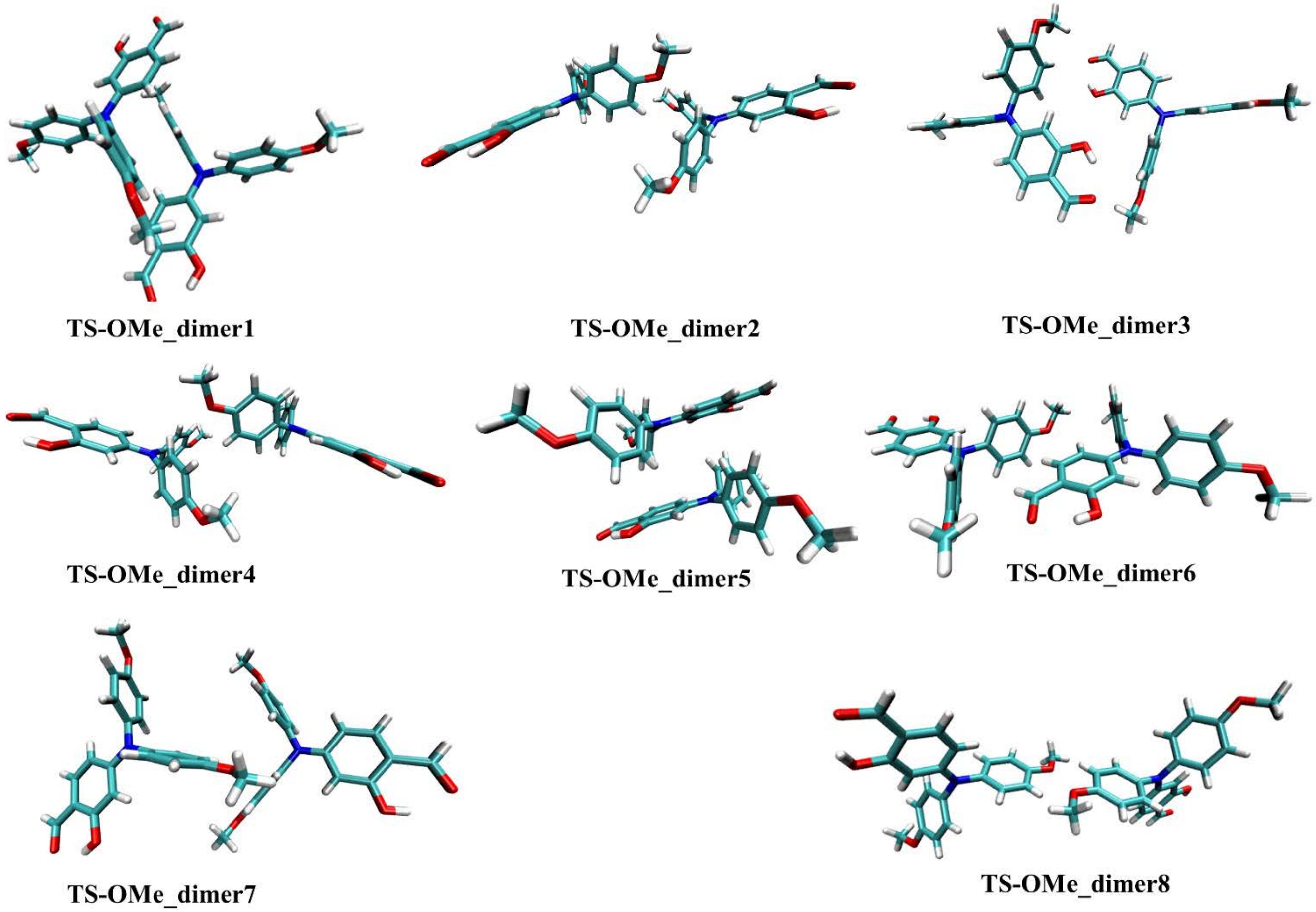

Fig. S3 The geometries of all the representative dimers of TS-OMe in crystal.

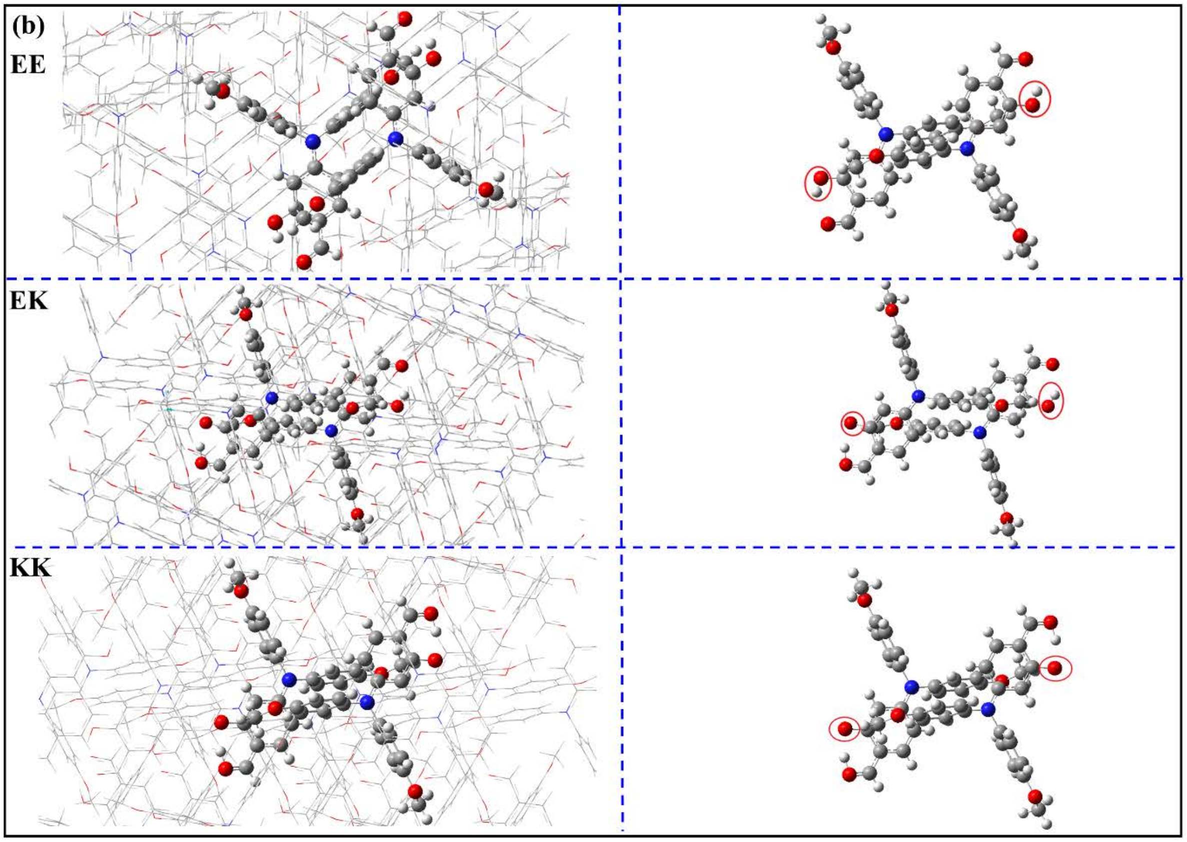

Fig. S4 Three different dimeric structures in the QM region optimized from ONIOM approach in crystal (a) TS and (b) TS-OMe.

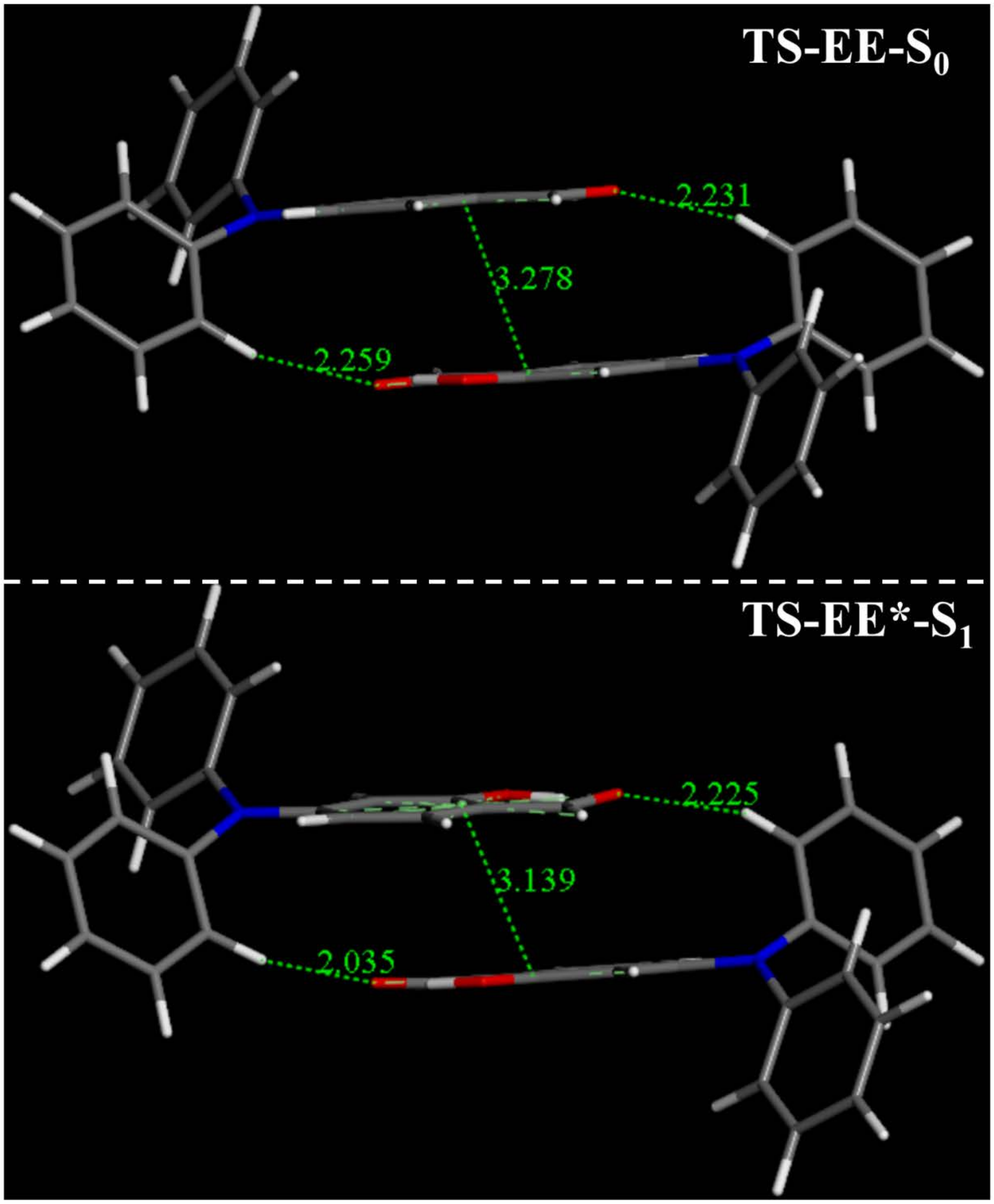

Fig. S5 Calculated C-H···O hydrogen bonds and π···π interactions with signed distances (Å) between the two E-formed TS monomers within the EE (upper panel in S_0 state) and EE* (lower panel in S_1 state) dimers in the QM region.
